# Flotillin-2 promotes cell proliferation via activating the c-Myc/BCAT1 axis by suppressing miR-33b-5p in nasopharyngeal carcinoma

**DOI:** 10.18632/aging.202726

**Published:** 2021-03-19

**Authors:** Rong Liu, Jie Liu, Ping Wu, Hong Yi, Bin Zhang, Wei Huang

**Affiliations:** 1 Department of Otolaryngology Head and Neck Surgery, Xiangya Hospital, Central South University, Changsha 410008, China; 2 College of Biology and Environmental Sciences, Jishou University, Jishou 416000, China; 3 Department of Pathology, Changsha Central Hospital, Changsha 410004, China; 4 Research Center of Carcinogenesis and Targeted Therapy, Xiangya Hospital, Central South University, Changsha 410008, China; 5 Department of Histology and Embryology, School of Basic Medicine, Central South University, Changsha 410013, China

**Keywords:** nasopharyngeal carcinoma, FLOT2, BCAT1, c-Myc, proliferation

## Abstract

Previously, we elucidated the function of flotilin-2 (FLOT2) and branched-chain amino acid transaminase 1(BCAT1) in nasopharyngeal carcinoma (NPC). However, the relationship between FLOT2 and BCAT1 in promoting NPC progression remains unknown. Here, we observed that FLOT2 upregulated BCAT1 expression in NPC cells. Ectopic expression of BCAT1 significantly antagonized the inhibitory effects on NPC cell proliferation induced by FLOT2 depletion. Consequently, BCAT1 knockdown markedly inhibited the pro-proliferative effects of FLOT2 overexpression in NPC cells. FLOT2 expression was positively correlated with BCAT1 expression in NPC tissues and was inversely correlated with the prognosis of NPC patients. Mechanistically, FLOT2 maintains the expression level of c-Myc, a positive transcription factor of BCAT1, and subsequently promote BCAT1 transcription. FLOT2 inhibited miR-33b-5p in NPC cells and attenuated its inhibitory effects on c-Myc. Further, experimental validation of the function of the FLOT2/miR-33b-5p/c-Myc/BCAT1 axis in regulating NPC cell proliferation was performed. Our results revealed that FLOT2 promotes NPC cell proliferation by suppressing miR-33b-5p, to maintain proper levels of c-Myc, and upregulate BCAT1trancription. Therefore, the FLOT2/miR-33b-5p/c-Myc/BCAT1 axis is a potential therapeutic target for NPC.

## INTRODUCTION

Nasopharyngeal carcinoma (NPC) is one of the most common head and neck cancer, that is highly prevalent in southern China and Southeast Asia [1, 2]. Radiation therapy is the primary treatment option, with a high cure rate in early-stage NPC [1, 3]. Unfortunately, most NPC patients are diagnosed at advanced stages, characterized by peripheral invasion and lymph node and distant metastasis. Besides, advanced stage NPC patients lack distinguishable symptoms and early diagnostic markers. Radiotherapy and chemotherapy show synergistic effects on killing NPC cells, however, the treatment outcome of advanced-stage NPC patients is far from satisfactory indicating poor prognosis [1–4]. Thus, to reveal the molecular mechanism involved in the development and progression of NPC, and subsequently identify potential prognostic markers of NPC is of great significance.

FLOT2 is a lipid raft scaffold protein that is highly ordered membrane subdomains and plays a critical role in maintaining the homeostasis of lipid rafts and subsequently allows the lipid rafts to realize its regulatory effects in multiple processes, such as cell adhesion, signal transduction, protein sorting, and trafficking, and in all critical processes in tumorigenesis and malignant progression [5, 6]. Therefore, aberrant expression of FLOT2 leads to dysregulation of lipid rafts and its downstream processes, which are closely correlated with pathological conditions such as cancer [7–9]. Upregulation of FLOT2 predicts poor prognosis and promotes malignant progression, proliferation, migration, invasion, and metastasis, of multiple tumors, including breast, cervical, colorectal, stomach, liver, lung, glioma, melanoma, as well as NPC, etc. by activating oncogenic pathways such as NF-κB, PI3K/AKT, TGFβ, and MEK/Raf/ERK [9–13]. Numerous studies report the role of FLOT2 as an oncogene involved in the regulation of tumor-promoting signaling molecules and pathways. Besides, we also previously confirmed the upregulation and oncogenic roles of FLOT2 in NPC [13]. However, detailed mechanisms on these roles remain unknown and need further exploration.

BCAT1, a cytosolic branched-chain aminotransferase, catalyzes the conversion of branched-chain amino acids, including leucine, isoleucine, and valine, into their corresponding branched-chain a-keto acids (BCAAs) by transferring an amino group onto a-ketoglutarate to generate glutamate [14]. Numerous studies reveal that activating BCAT1 enhance the tumor-driving and -promoting roles of BCAAs metabolism in gliomas and myeloid leukemia [15–18]. Moreover, BCAT1 upregulation and its oncogenic role have been observed in other tumors such as hepatocellular, gastric, breast, ovarian, endometrial, prostate, and esophageal squamous cell carcinomas [14, 19–24]. Our previous study also demonstrated that the transcription factor c-Myc upregulates BCAT1 expression to promote tumor initiation and progression in NPC. Besides, Microarray analysis indicated that FLOT2 depletion significantly downregulated BCAT1 expression in NPC cells, suggesting that BCAT1 may be involved in the pro-tumor roles of FLOT2 in NPC.

Therefore, in the present study, we investigated the FLOT2 regulation of BCAT1 in NPC. The results demonstrated that FLOT2 activated BCAT1 through c-Myc regulation of BCAT1 transcription, thereby promoting NPC proliferation. miR-33b-5p, a direct negative regulator of c-Myc, was inhibited by FLOT2, subsequently suppressing c-Myc mRNA degradation, and promoting BCAT1 expression. Moreover, FLOT2, c-Myc, and BCAT1 were positively correlated in NPC tissues, and FLOT2/c-Myc/BCAT1 axis was positively correlated with NPC progression and negatively associated with the prognosis of NPC patients. Therefore, our findings suggest that FLOT2/miR-33b-5p/c-Myc/BCAT1 axis is a potential therapeutic target in NPC.

## RESULTS

### FLOT2 positively regulates BCAT1 expression in NPC cells

**Figure 1:**
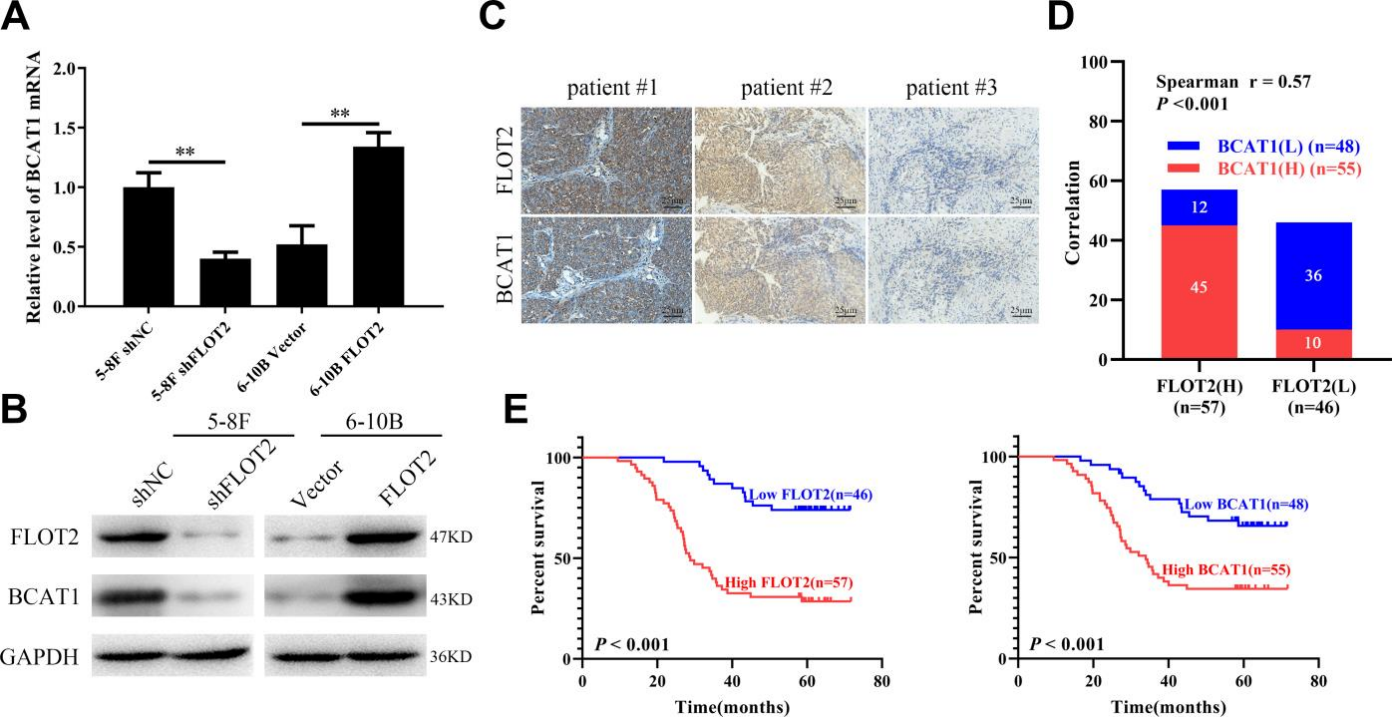
**FLOT2 positively regulates and predicts poor prognosis in NPC.** (**A**, **B**) qPCR and western blot assays indicating the level of BCAT1 mRNA and protein in 5-8F-shFLOT2, 6-10B-BCAT1 and control cells. (**C**) The representative IHC pictures indicating the co-expression patterns of FLOT2 and BCAT1 in patients with NPC (magnification, 200×). (**D**) Spearman correlation analysis indicating the positive correlation between FLOT2 and BCAT1 in NPC. (**E**) Kaplan-Meier survival analysis showing the level of FLOT2 and BCAT1 negatively associates with overall survival outcome in NPC.

Previously, we used cDNA microarray (GSE67456) to explore the effects of FLOT2 knockdown on the transcription profile in NPC cells [13]. The results showed that BCAT1 was downregulated in 5-8F-shFLOT2 cells. Thus, the expression of BCAT1 was further validated in NPC cells by altering the expression of FLOT2. qPCR and western blot analysis (Figure 1A, 1B) revealed that FLOT2 knockdown significantly decreased BCAT1 expression, while FLOT2 overexpression remarkably increased BCAT1 expression in 5-8F and 6-10B cells, respectively. FLOT2 expression levels in NPC cells were also determined by altering the expression of BCAT1 alteration. The results showed that both BCAT1 knockdown and overexpression had no effects on FLOT2 expression (Supplementary Figure 1).

### FLOT2 and BCAT1 are positively correlated in NPC and predicts poor prognosis of NPC patients

The correlation between FLOT2 and BCAT1 was explored, as well as their clinical and prognostic value in NPC cells. IHC staining showed that FLOT2 expression had a positive correlation with BCAT1 expression in NPC cells (r = 0.57) (Figure 1C, 1D). Moreover, FLOT2 and BCAT1 expression were positively correlated with several pathological variables, including T, N, M, and TNM stages (Table 1). Besides, FLOT2 and BCAT1 expression levels were negatively correlated with the OS of NPC patients (Figure 1E). Furthermore, univariate Cox proportional hazards regression analysis revealed that primary T stage, TNM stage, and FLOT2 and BCAT1 expression levels were significantly related to the OS of NPC patients (Table 2). Multivariate Cox proportional hazards regression analysis demonstrated that FLOT2 and BCAT1 were independent predictors of reduced OS of NPC patients (Table 2). Therefore, these results indicate that the FLOT2 and BCAT1 axis play some important roles in NPC progression and poor prognosis in patients with NPC.

**Table 1:** Correlations between FLOT2/BCAT1 level and clinicopathological characteristics in NPC (N=103, χ^2^ test).

**Variables**	**N**	**FLOT2**				**BCAT1**		
		**Low**	**High**	***P***		**Low**	**High**	***P***
**Gender**								
Male	54	25	29	0.440		27	27	0.299
Female	49	21	28			21	28	
**Age**								
≤ 45	40	19	21	1.206		20	20	0.364
>45	63	27	36			28	35	
**Primary T stage**								
T1-2	29	18	11	0.030		17	12	0.095
T3-4	74	28	46			31	43	
**Lymph node (N) metastasis**								
N0	44	26	18	0.016		23	21	0.213
N1-3	59	20	29			25	34	
**M stage**								
M0	77	41	36	0.002		43	34	0.001
M1	26	5	21			5	21	
**TNM stage**								
I-II	42	27	15	0.001		25	17	0.029
III-IV	61	19	42			23	38	
**Smoking History**								
Yes	28	14	14	0.328		13	15	0.982
NO	75	32	43			35	40	

**Table 2:** Univariate and multivariate analyses of selected prognostic factors for overall survival using Cox proportional hazards regression model (N=103).

**Variables**	**Overall survival (OS)**			
	**Univariate analysis**		**Multivariate analysis**	
	***P***	**HR (95%CI)**	***P***	**HR (95%CI)**
**Age**				
≤45 *vs*. >45	0.704	0.896 (0.509-1.577)	0.220	0.467 (0.244-0.897)
**Gender**				
Male *vs.* Female	0.911	1.032 (0.598-1.781)	0.240	0.668 (0.341-1.309)
**Primary tumor(T) stage**				
T1-2 *vs.*T3-4	**< 0.001**	4.872 (2.471-9.109)	0.050	1.316 (1.099-3.012)
**lymph node(N) metastasis**				
N0 *vs.*N1-3	**0.001**	2.909 (1.549-5.464)	0.080	1.090 (0.549-2.167)
**Distant metastasis(M)**				
M0 *vs*. M1	**< 0.001**	2.366 (1.270-4.097)	**0.004**	0.326 (0.153-0.696)
**Clinical TNM stage**				
I-II *vs.* III-IV	**< 0.001**	1.656 (1.005-3.281)	**< 0.001**	1.050 (1.013-2.189)
**Smoking history**				
Yes *vs*. No	0.615	1.166 (0.640-2.125)	0.226	0.620 (0.286-1.343)
**FLOT2 level**				
High *vs*. Low	**< 0.001**	2.692 (1.448-3.995)	**0.002**	2.184 (1.062-3.545)
**BCAT1 level**				
High *vs*. Low	**< 0.001**	2.893 (1.600-5.230)	**0.037**	1.565 (0.577-4.246)

### FLOT2 promotes cell proliferation both *in vitro* and *in vivo* via BCAT1 in NPC

**Figure 2:**
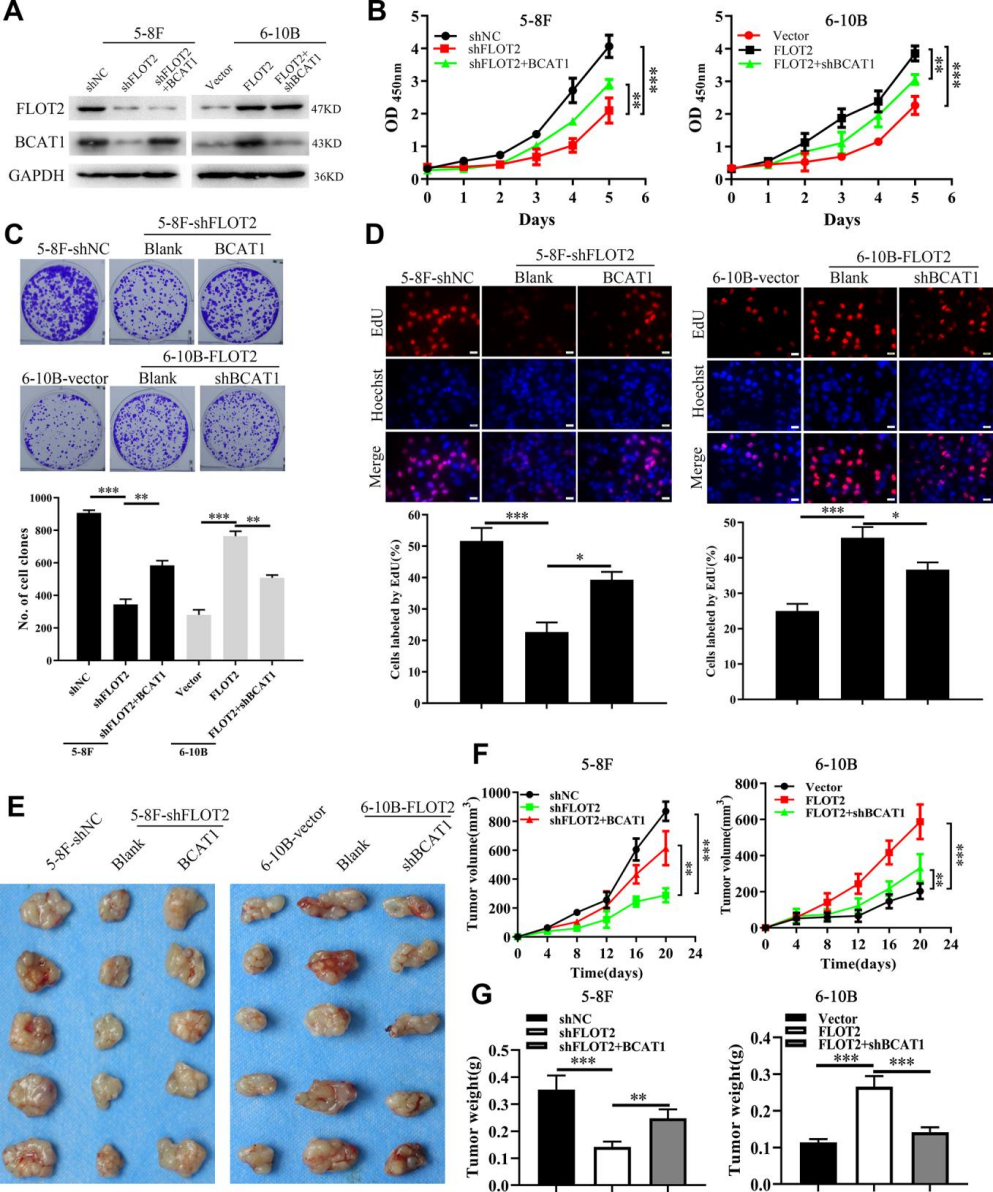
**FLOT2 promotes proliferation via positively regulating BCAT1 in NPC.** (**A**) Western blot assay indicating the level of FLOT2 and BCAT1 in 5-8F-shFLOT2, 5-8F-shFLOT2+BCAT1, 6-10B-FLOT2, 6-10B-FLOT2+shBCAT1, and control cells. (**B**–**D**) CCK-8, plate clone formation, and EdU assays showing the growth and proliferation abilities of 5-8F-shFLOT2, 5-8F-shFLOT2+BCAT1, 6-10B-FLOT2, 6-10B-FLOT2+shBCAT1, and control cells *in vitro*. 5-8F-shFLOT2, 5-8F-shFLOT2+BCAT1, 6-10B-FLOT2, 6-10B-FLOT2+shBCAT1, and control cells were subcutaneously implanted into nude mice (n = 5 each group) for xenografts formation to analyze the proliferation *in vivo.* The pictures (**E**), the volumes (**F**), and weights (**G**) of xenografts were presented. *, *P* <0.05, **, *P* < 0.01, ***; *P* < 0.001.

Next, we explored whether BCAT1 mediated FLOT2 function in NPC. Firstly, western blot analysis successfully demonstrated ectopic expression of BCAT1 in 5-8F-shFLOT2 cells and BCAT1 depletion in 6-10B-FLOT2 cells (Figure 2A). *In vitro* and *in vivo* experiments determined the effects of BCAT1 alterations on cell proliferation. CCK-8, plate clone formation, and EdU incorporation assays revealed that BCAT1 restoration significantly rescued the inhibitory effects of FLOT2 knockdown on 5-8F cell proliferation (Figure 2B, left arm; 2C upper arm; 2D left arm). Depletion of BCAT1, antagonized the pro-proliferation effects of FLOT2 overexpression on 6-10B cells (Figure 2B, right arm; 2C low arm; 2D right arm). Consequently, ectopic expression of BCAT1 remarkably removed the inhibition of FLOT2 silencing on subcutaneous growth of 5-8F cells (Figure 2E–2G, left arm); while BCAT1 knockdown significantly counteracted the activation of FLOT2 overexpression on subcutaneous growth of 6-10B cells (Figure 2E–2G, right arm). Therefore, these results indicated that FLOT2 promoted the proliferation of NPC cells via positive regulation of BCAT1 both *in vitro* and *in vivo*.

### The pro-proliferation effect of BCAT1 depends on its enzymatic activity in NPC

**Figure 3:**
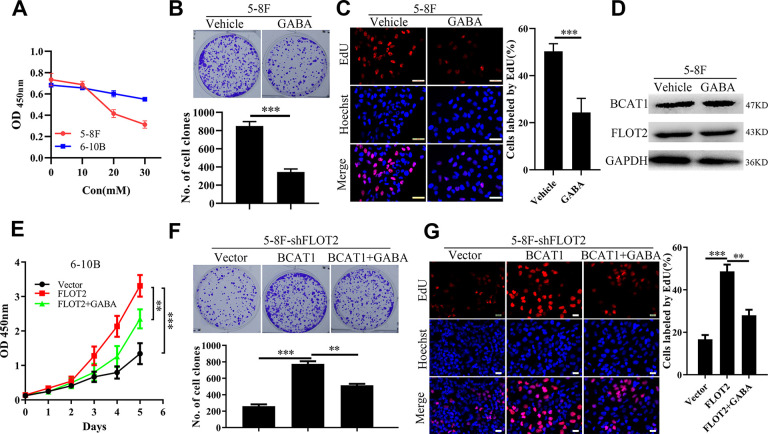
**GABA antagonizes the pro-proliferative effects of BCAT1 in NPC.** (**A**) CCK-8 assay indicating the effects of GABA at different concentration on 5-8F and 6-10B cells. (**B**, **C**) plate clone formation and EdU assays showing the inhibitory effects of GABA at 20mM on proliferation of 5-8F cells. (**D**) Western blot demonstrating the levels of BCAT1 and FLOT2 in 5-8F cells treated by GABA at 20mM. (**E**–**G**) CCK-8, plate clone formation, and EdU assays showing inhibitory effects on 6-10B-FLOT2 cells treated by GABA at 20mM. **, *P* < 0.01, ***; *P* < 0.001.

BCAT1 function depends on its enzymatic activity [17]. Therefore, BCAT1 activity was inhibited with gabapentin (GABA), a γ-aminobutyric acid analogue and a competitive inhibitor of BCAT1, to determine its effects on NPC cell proliferation. Firstly, NPC cell viability was measured using CCK-8 assay, at different gabapentin concentrations. As shown in Figure 3A, GABA inhibited the growth of 5-8F cells expressing high levels of BCAT1, in a dose-dependent manner, but exerted little influence on the growth of 6-10B cells expressing low levels of BCAT1. We further explored the inhibitory effects of GABA on NPC cell proliferation. Consistently, the inhibitory effects of GABA on NPC cell proliferation were further supported by results of the plate clone formation (Figure 3B) and EdU incorporation assays (Figure 3C). Besides, GABA did not influence the protein expression level of BACT1 and FLOT2 (Figure 3D). However, CCK-8 (Figure 3E), plate clone formation (Figure 3F), and EdU incorporation assays (Figure 3G) showed that GABA treatment significantly antagonized the rescue effects of BCAT1 on 5-8F-shFLOT2 cell proliferation. Taken together, these results suggested that the pro-proliferation role of BCAT1 highly depends on its enzymatic activity in NPC.

### FLOT2 upregulates BCAT1 and promotes NPC cell proliferation via c-Myc regulation

**Figure 4:**
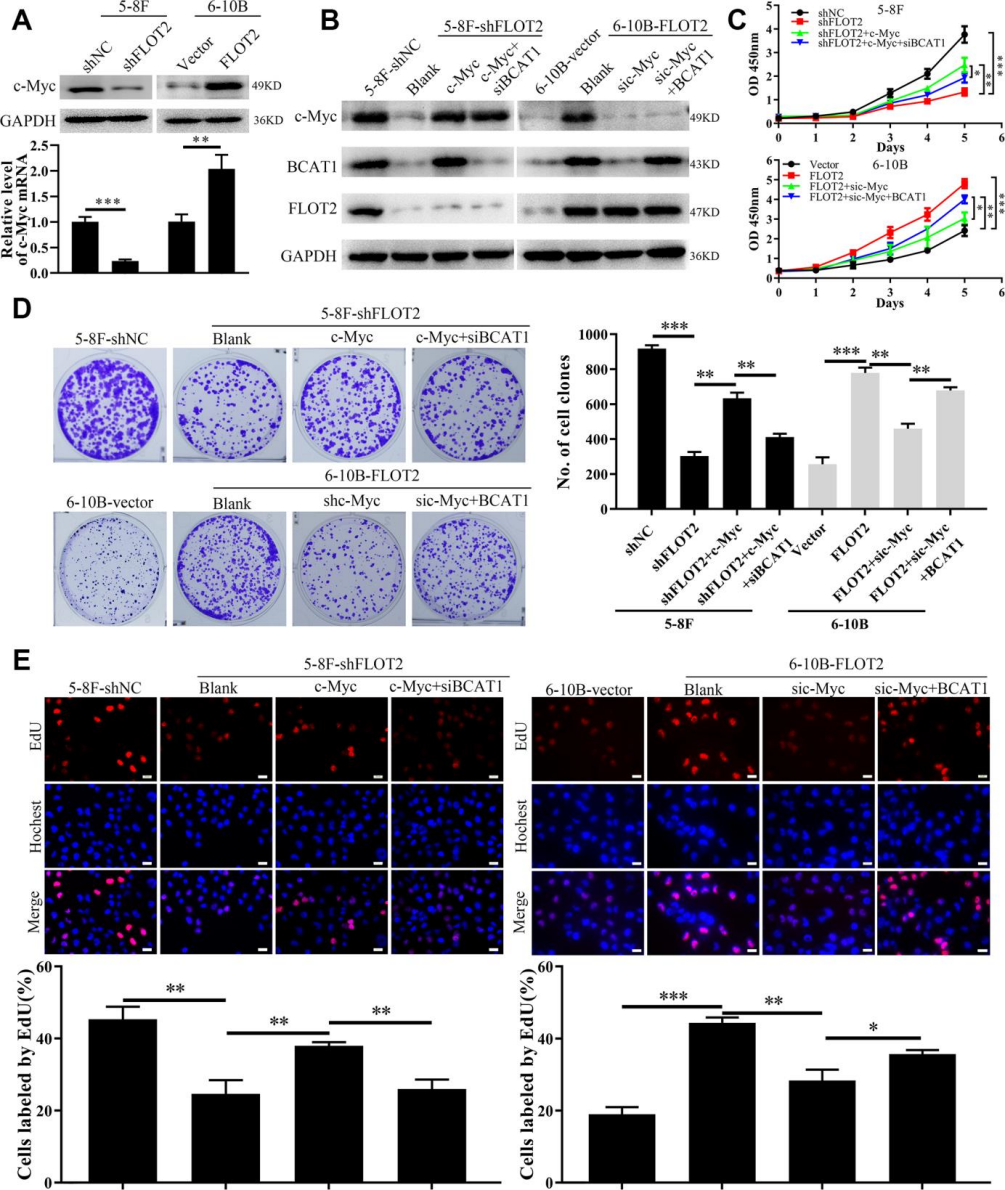
**FLOT2 regulates BCAT1 and promotes cell proliferation via maintaining c-Myc in NPC.** (**A**) Western blot and qPCR assays indicating the level of c-Myc mRNA and protein in 5-8F-shFLOT2, 6-10B-BCAT1 and control cells. (**B**) Western blot showing the level of c-Myc, BCAT1 and FLOT2 in 5-8F-shFLOT2, 5-8F-shFLOT2+c-Myc, 5-8F-shFLOT2+c-Myc+siBCAT1, 6-10B-BCAT1, 6-10B-FLOT2+sic-Myc, 6-10B-FLOT2+sic-Myc+BCAT1, and control cells. (**C**–**E**) CCK-8, plate clone formation, and EdU assays showing the growth and proliferation abilities of 5-8F-shFLOT2, 5-8F-shFLOT2+c-Myc, 5-8F-shFLOT2+c-Myc+siBCAT1, 6-10B-BCAT1, 6-10B-FLOT2+sic-Myc, 6-10B-FLOT2+sic-Myc+BCAT1, and control cells. *, *P* <0.05, **, *P* < 0.01, ***; *P* < 0.001.

Our previous study confirmed that c-Myc promoted BCAT1 transcription in NPC cells [25]. Therefore, in the current study, we explored whether FLOT2 increased BCAT1 expression via c-Myc. Indeed, both mRNA and protein expression levels of c-Myc were downregulated in 5-8F-shFLOT2 cells and upregulated in 6-10B-FLOT2 cells (Figure 4A), respectively. Moreover, ectopic expression of c-Myc restored BCAT1 expression which was decreased by siBCAT1 in 5-8F-shFLOT2 cells (Figure 4B, left arm). c-Myc knockdown in 6-10B-FLOT2 downregulated BCAT1 expression levels, which was restored by ectopic expression of BCAT1 (Figure 4B, right arm). Similarly, functional experimental results revealed that the proliferative capacity of 5-8F-shFLOT2 cells was restored by ectopic expression of c-Myc (Figure 4C, upper arm; 4D, 4E, left arm), which was suppressed by BCAT1 knockdown. c-Myc knockdown inhibited 6-10B-FLOT2 cell proliferation, which was restored after ectopic expression of BCAT1 (Figure 4C, low arm, 4D, 4E, right arm). Furthermore, in our previous study, we investigated the effects of BCAT1 and c-Myc depletion on AKT and NF-κB activity, which mediates FLOT2 oncogenic role in NPC [13]. Both BCAT1 and c-Myc knockdown significantly inhibited AKT and NF-κB activity, which was confirmed by a decreased in p-AKT and p-p65 expression (Supplementary Figure 2A) in NPC cells. Considering that inhibition of AKT and NF-κB activity by MK2206 and BAY 11–7082, respectively, exert no notable effects on the expression levels of BCAT1 and c-Myc (Supplementary Figure 2B), suggests that AKT and NF-κB could serve as the downstream signaling of c-Myc/BCAT1 in mediating the functions of FLOT2 in NPC. Therefore, these results demonstrated that FLOT2 upregulated BCAT1 and promoted NPC cell proliferation via positive regulation of c-Myc.

### FLOT2 upregulates c-Myc by inhibiting miR-33b-5p expression in NPC cells

**Figure 5:**
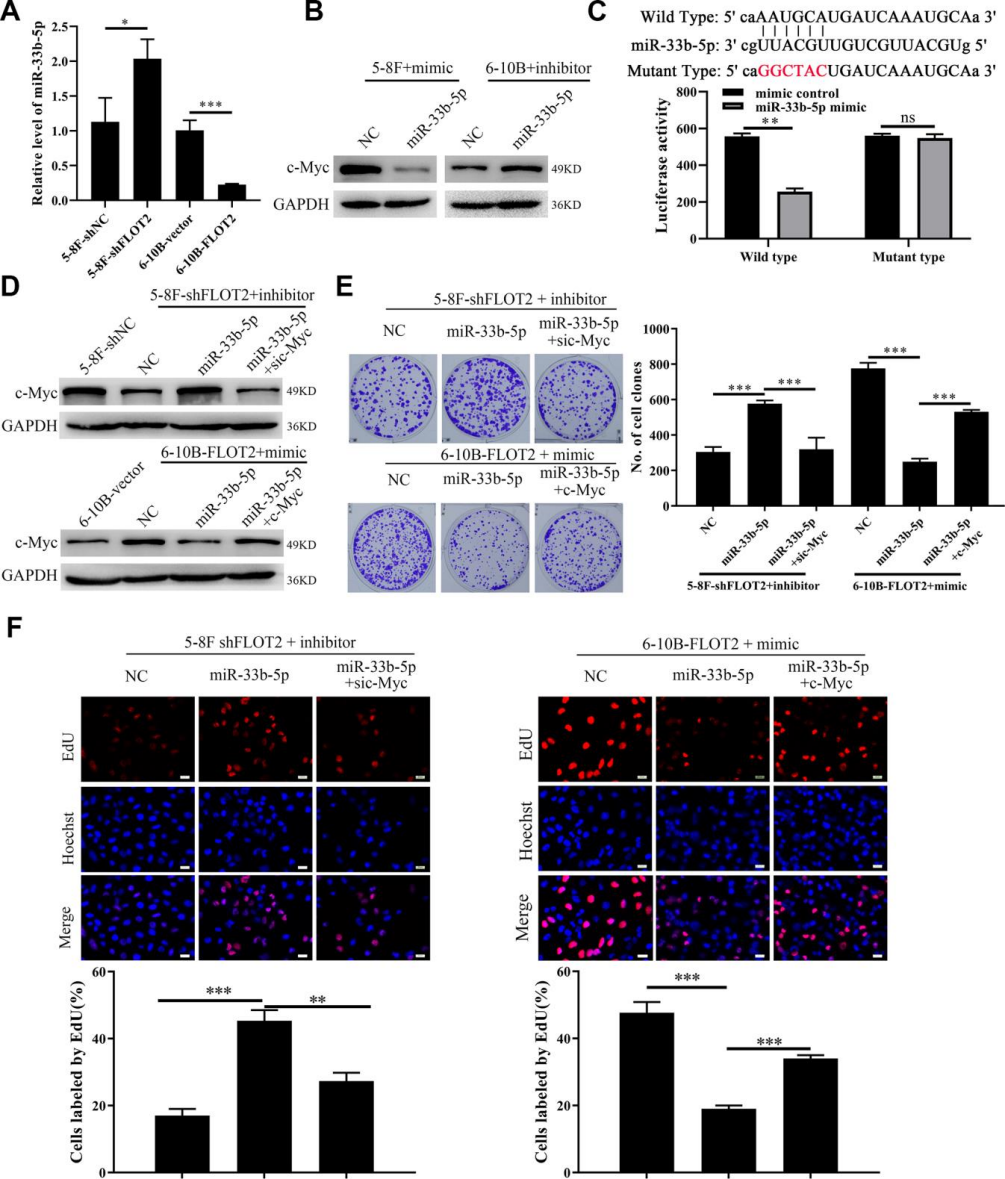
**FLOT2 regulates c-Myc and promotes cell proliferation via inhibiting miR-33b-5p in NPC.** (**A**) qPCR assay demonstrating the relative expression of miR-33b-5p in 5-8F-shFLOT2, 6-10B-BCAT1 and control cells. (**B**) Western blot showing the level of c-Myc in 5-8F and 6-10B cells transfected with miR-33b-5p mimic and inhibitor, respectively. (**C**) Luciferase activities of 5-8F cells co-transfected with miR-33b-5p+wild type c-Myc and miR-33b-5p+mutant type c-Myc, respectively. (**D**) Western blot showing the level of c-Myc in 5-8F-shFLOT2, 5-8F-shFLOT2+miR-33b-5p inhibitor, 5-8F-shFLOT2+miR-33b-5p inhibitor+sic-Myc, 6-10B-FLOT2, 6-10B-FLOT2+miR-33b-5p mimic, 6-10B-FLOT2+miR-33b-5p mimic+c-Myc and control cells. (**E**, **F**) Plate clone formation and EdU assays showing the growth and proliferation abilities of 5-8F-shFLOT2, 5-8F-shFLOT2+miR-33b-5p inhibitor, 5-8F-shFLOT2+miR-33b-5p inhibitor+sic-Myc, 6-10B-FLOT2, 6-10B-FLOT2+miR-33b-5p mimic, 6-10B-FLOT2+miR-33b-5p mimic+c-Myc cells. *, *P* <0.05, **, *P* < 0.01, ***; *P* < 0.001.

We explored the molecular mechanism of FLOT2 regulation of c-Myc in NPC cells. Generally, both transcriptional and post-transcriptional mechanisms can lead to fluctuation of mRNA levels [26]. Therefore, qPCR was used to detect the expression of c-Myc hnRNA in FLOT2 knockdown NPC cells using two sets of specific primers amplifying intron-1 and -2, respectively. We found that the knockdown of FLOT2 had no significant effect on the expression of c-Myc hnRNA in NPC cells (Supplementary Figure 3A), suggesting that FLOT2 mediates the post-transcriptional mechanism of c-Myc regulation in NPC cells. Moreover, MG132, a proteasome inhibitor, could not rescue the down-regulation of c-Myc (Supplementary Figure 3B), indicating that FLOT2 does not affect c-Myc stability. Therefore, we further investigated whether miRNAs, the most common post-transcriptional regulators, mediated the regulation of c-Myc in NPC cells through FLOT2. We predicted the candidate miRNAs targeting c-Myc via ENCORI (The Encyclopedia of RNA Interactomes) database. miR-33b-5p, a tumor suppressor gene, which directly inhibits c-Myc in several cancers, was selected for validation [27–30]. Indeed, silenced FLOT2 upregulated the miR-33b-5p level in 5-8F cells, while FLOT2 overexpression downregulated miR-33b-5p expression in 6-10B cells (Figure 5A). Besides, miR-33b-5p mimic significantly downregulated c-Myc in 5-8F cells, while miR-33b-5p inhibitor upregulated c-Myc in 6-10B cells (Figure 5B). Moreover, miR-33b-5p significantly inhibited luciferase activity of 5-8F cells transfected with wild-type c-Myc plasmids (Figure 5C), confirming that miR-33b-5p directly targeted c-Myc in NPC cells. Consequently, miR-33b-5p inhibitors restored c-Myc levels in 5-8F-shFLOT2 cells (Figure 5D, upper arm), and miR-33b-5p mimics reduced the level of c-Myc in 6-10B-FLOT2 cells (Figure 5D, low arm). miR-33b-5p inhibitors restored 5-8F-shFLOT2 cell proliferation, which was suppressed by c-Myc knockdown (Figure 5E, upper arm, Figure 5F, left arm). miR-33b-5p mimics, on the other hand, inhibited 6-10B-FLOT2 cell proliferation, which was rescued by ectopic expression of c-Myc (Figure 5E, low arm, Figure 5F, right arm). Our results indicated that FLOT2 activates c-Myc/BCAT1 and promotes NPC cell proliferation through suppressing miR-33b-5p.

## DISCUSSION

Previously, we elucidated the pro-neoplastic role of FLOT2 and BCAT1 in NPC [13, 25, 31]. In this study, we explored the relationship between FLOT2 and BCAT1 and determined that FLOT2 upregulated BCAT1 expression, thereby promoting NPC cell proliferation. Mechanistically, we found that FLOT2 suppressed miR-33b-5p levels and target inhibition of miR-33b-5p on c-Myc, and upregulated BCAT1 expression. The results reveal that FLOT2/miR-33b-5p/c-Myc/BCAT1 is an oncogenic axis that is of potential clinical significance in NPC treatment.

Current studies have established the oncogenic roles of FLOT2 in cancers. Upregulation of FLOT2 promotes the proliferation, differentiation, migration, invasion, metastasis, and therapy resistance of cancer, and positively correlates with poor prognosis in patients with cancer [10, 12, 13, 32]. The activation of signaling pathways, such as NF-κB, Raf/MEK/ERK1/2, TGF-β, and PI3K/AKT, drive process including suppression of apoptosis, cell cycle acceleration, and epithelial-mesenchymal transition (EMT), which accounts for mechanisms underlying the oncogenic function of FLOT2 [8, 9, 11–13, 33]. However, detailed mechanisms of how FLOT2 activates oncogenic signaling and other mechanisms mediating the functions of FLOT2, need to be further investigated. A recent study indicated that PLCD3 is directly associated with FLOT2 to promote the progression of NPC cells, however, the study failed to illustrate the specific role of PLCD3 in FLOT2’s pro-neoplastic function in NPC [34]. In this study, we revealed that FLOT2 upregulated BCAT1 expression and activity, and in turn promoted the malignant progression of NPC, thus, presenting a new oncogenic mechanism of FLOT2. Besides, the results suggested that BCAA metabolism is involved in NPC development.

Dysregulation of BCAA metabolism contributes to tumorigenesis and progression in multiple cancers [14]. Besides, aberrant activation and upregulation of BCAT1, a critical enzyme in BCAA metabolism, serves as the main underlying molecular mechanism of reprogramming BCAA metabolism in cancer. BCAT1 overexpression enhances BCAA catabolism, which subsequently promotes the proliferation and stemness of cancerous cells in gliomas with wild-type IDH1, myeloid leukemia, and endometrial cancer [15–18, 35]. Besides, BCAT1 overexpression is reported in other cancers, where BCAT1 functions as an oncogene, stimulates malignant progression and is negatively correlated with the prognosis of patients [14, 19, 20, 22–24]. Our previous study revealed that BCAT1 overexpression promotes cell proliferation, migration, and invasion in NPC [25, 31]. Consistently, this study confirmed the oncogenic role of BCAT1 in NPC and further revealed that BCAT1 was an independent marker for poor prognosis of NPC patients.

Both transcriptional and post-transcriptional mechanisms are involved in the regulation of BCAT1 expression. Transcription factors HIF-1α and c-Myc, DNA methylation status, and miRNAs, such as miR-124-3p, miR-203, miR-218, miR-503, are reported to regulate the transcription and mRNA stability of BCAT1 in cancers, respectively [21, 23, 36–39]. Based on our previous findings that c-Myc regulates BCAT1 transcription in NPC [25], we explored whether FLOT2 increases BCAT1 expression in a c-Myc dependent manner in NPC. The results showed that FLOT2 sustained the c-Myc level in NPC cells by suppressing miR-33b-5p, a known miRNA that directly targets c-Myc. Previous studies indicate that FLOT2 regulation of BCAT1 expression in a c-Myc dependent manner inhibits the progression of prostate cancer and osteosarcoma [27, 28]. The tumor-suppressive roles of miR-33b-5p have also been reported in other cancers including gastric cancer, colorectal cancer, and renal cell carcinoma [40–42]. The current study also confirmed the anti-tumor functions of miR-33b-5p in NPC.

However, we failed to reveal the regulatory mechanism of miR-33b-5p targeting FLOT2 in NPC. Snail1, SREBF1, and lncRNAs (long coding RNAs) are known regulators of miR-33b-5p [43–47]. Snail1 maintains the function of cancer-associated fibroblasts and induces EMT of lung cancer cells by suppressing miR-33b [43]. Our previous study confirmed that FLOT2 knockdown significantly decreased Snail level in NPC cells [13], suggesting that FLOT2 suppresses miR-33b-5p through the sustained expression of Snail1 in NPC, however, these findings will be validated through additional future experiments.

In conclusion, our study reveals that FLOT2 suppresses miR-33b-5p, subsequently attenuating the inhibitory effects of miR-33b-5p on c-Myc, leading to BCAT1 upregulation to promote NPC cell proliferation. These findings suggest that FLOT2/miR-33b-5p/c-Myc/BCAT1 axis is a potential therapeutic target in NPC.

## MATERIALS AND METHODS

### Patients and tissue samples

103 cases of paraffin-embedded and poorly differentiated squamous NPC tissue samples were collected from primary patients without any therapy during Jan 2010 to Dec 2012 in Xiangya Hospital of Central South University. The clinical stage of the patients was classified according to the seventh edition of AJCC guidelines as our previous illustration [26]. The patients uniformly received radiochemotherapy and the clinicopathological features, prognostic conditions of patients were retrospectively collected. Overall survival (OS) was defined as the time from the initiation of primary therapy to the date of cancer-related death or when censured at the latest date if patients were still alive.

### Cell lines

Human NPC cell lines, 5-8F and 6-10B, were cultured with RPMI-1640 medium (BI, Jerusalem, Israel) supplemented with 10% fetal bovine serum (BI, Jerusalem, Israel) as our previous description [13, 26]. Stable cell lines, including 5-8F-shFLOT2, 5-8F-shBCAT1, 6-10B-FLOT2 and control cell lines, were previously established by us [13, 25, 31]. 5-8F-shFLOT2+BCAT1 and 6-10B-FLOT2+shBCAT1 cell lines were constructed by infecting 5-8F-shFLOT2 and 6-10B-FLOT2 cells with lentivirus particles expressing BCAT1 and shBCAT1 (short hair BCAT1), respectively. The lentivirus expression plasmid pLV-puro-BCAT1 and pLV-U6-shBCAT1 were used to package lentiviral particles as our previous description [26]. MK2206 (Selleck, TX, USA) and BAY 11-7082 (Selleck, TX, USA) were added into the culture medium to inhibit the activities of AKT and NF-κB as indicated.

### Plasmids, siRNAs and miRNAs transfection

Plasmids, including pCMV-HA/c-Myc, pENTER-BCAT1 and control vectors, siRNAs (small inferring RNAs), including sic-Myc, siBCAT1 and siNC, miRNAs, including miR-33b-5p mimic, miR-33b-5p inhibitor and control miRNAs, were transfected into the corresponding cells as mentioned in manuscript using HighGene Transfection reagent (Abclonal, Wuhan, China) according to our previous description [26]. The siRNAs and miRNAs were purchased from Ribobio Inc. (Guangzhou, China). The sequences of siRNAs were listed as follow: si-c-Myc: 5’- AGACTCTGACACTGTCCA-3’, si-BCAT1: 5’-GAGCCCAGTGGGACCTTATTT-3’. The sequences of miRNAs were not offered by manufacturer.

### Western blot

Western blot was carried out as described previously by us [3, 26]. Briefly, the total proteins were collected from RIPA cell lysis by centrifugation. The proteins were denatured and separated by SDS-PAGE and subsequently transmitted into 0.22μm PVDF membrane. The membrane was blocked with Protein Free Rapid Blocking Buffer (Epizyme, Shanghai, China) for 20 minutes and subjected for antibody incubation overnight at 4° C. The follow antibodies were applied, including incubated with Rabbit anti-Flotillin-2(C42A3) (#3436, dilution 1:1000, CST, MA, USA), rabbit anti-BCAT1 antibody (D121976, dilution 1:200, BBI, Shanghai, China), rabbit anti-c-Myc (A1309, dilution 1:500, Abclonal, Wuhan, China), rabbit anti-p-AKT (Ser473)(D9E) (#4060, dilution 1:1000, CST, MA, USA), rabbit anti-AKT (#9272, dilution 1:1000, CST, MA, USA), rabbit anti-p-NF-κB p65 (Ser536)(93H1) (#3033, dilution 1:1000, CST, MA, USA), rabbit anti-NF-κB p65(D14E12) (#8242, dilution 1:1000, CST, MA, USA), and rabbit anti-GAPDH (AB-P-R001, dilution 1:1000, Goodhere, Hanzhou, China). Next day, after incubated with anti-rabbit or anti-mouse IgG HRP-conjugated secondary antibodies (D110058 or D110098, dilution 1:3000, BBI, Shanghai, China), the protein levels were visualized by chemiluminescent HRP substrate (EpiZyme, Shanghai, China).

### RNA isolation and quantitative real-time polymerase chain reaction(qPCR)

The RNA extraction and qPCR assay were carried out according to our previous reports [8, 12]. Briefly, total RNAs were extracted from the indicated NPC cells with Trizol reagent (Thermo Fischer Scientific). FastKing gDNA Dispelling RT SuperMix Kit (TIANGEN, Beijing, China) and miDETECT A TrackTM miRNA qRT-PCR Starter Kit (RiboBio, Guangzhou, China) were used to reversely transcribe the total RNA to cDNA as templates for mRNAs and miRNAs level detection, respectively. The relative levels of BCAT1, c-Myc, hnc-Myc and miR-33b-5p were analyzed by PCR using RealUniversal Color PreMix (SYBR Green) (TIANGEN, Beijing, China), according to the manufacturer’s instructions. The relative expressions of genes and miRNAs were quantified by using 2^-ΔΔCt^ method with 5S and GAPDH as internal controls. The primer sequences were summarized in the Supplementary Table 1.

### CCK-8

Cell proliferation was analyzed using a CCK-8 kit (Beyotime, Shanghai, China) as our previous description [3, 26]. The assay was performed three times in triplicate.

### Plate clone formation

Plate colony formation assay was carried out according to our previous description [26]. The assay was applied three times in triplicate.

### 5-ethynyl-2′-deoxyuridine (EdU) incorporation assay

EdU incorporation assay was performed to detect cell proliferation as described previously by us [26]. The assay was performed three times in triplicate.

### Immunohistochemistry (IHC)

The level of FLOT2 and BCAT1 in NPC tissues were detected with SP Rabbit & Mouse HRP Kit (ZSGB-BIO, Beijing, China) as previously described by us [26]. Simply, after antigen retrieval, removal of endogenous peroxidase activity, and blocking, tissue sections were incubated with rabbit anti-FLOT2 antibody (D225408, dilution 1:50, BBI, Shanghai, China) and rabbit anti-BCAT1 antibody (D121976, dilution 1:200, BBI, Shanghai, China) overnight at 4° C. Next, the sections were incubated with biotinylated secondary antibody followed by avidin-biotin peroxidase complex. Finally, tissue sections were stained with 3′, 3′-diaminobenzidine (DAB, ZSGB-BIO, Beijing, China) and counterstained with hematoxylin. Immunohistochemical staining was independently evaluated by two pathologists. The protein expressions were scored based on the staining intensity and area. The percentage of stained cells was categorized as no staining = 0, < 30% of stained cells = 1, 30~60% = 2, and > 60% = 3. The evaluation of staining intensity was as follow: absent staining as 0, weak as 1, moderate as 2, and strong as 3. The staining score (ranging from 0-6) for each tissue was calculated by adding the area score and the intensity score. A combined staining score of ≤3 was considered to be low expression, and > 3 was considered to be high expression.

### Dual-luciferase reporter system assay

Dual-luciferase reporter system assay was performed as our previous description [26, 48]. Simply, as Figure 5C indicating, c-Myc wild 3′-UTR and mutant 3′-UTR luciferase reporter plasmids were co-transfected with miR-33b-5p mimic or mimic control into 5-8F cells HighGene Transfection reagent (Abclonal, Wuhan, China). 48 hour later, both firefly luciferase and renilla luciferase activities were detected by using the dual-luciferase reporter assay system (Promega, WI, USA).

### *In vivo* proliferation assay

*In vivo* proliferation assay was analyzed as our previous illustration [26, 48]. Briefly, female nude Balb/c mice, 4 weeks old, were purchased from the Laboratory Animal Center of Central South University (Changsha, China) and were maintained under specific pathogen-free conditions. For tumor formation experiment, 2×10^6^ cells were subcutaneously injected into the flanks of mice (n=5 mice each). The length and width of xenografts were detected with caliper every 4 days. Tumor volume (in mm3) was calculated by using the modified ellipse formula (volume = length × width2/2). 20 days later, the mice were killed by cervical dislocation, and their tumors were excised and weighted.

### Ethics statement

The usage of human tissues was approved by the Ethics Committee of Xiangya Hospital, Central South University. Considering only archived tumor specimens were enrolled in this study, the ethics committee waived the need for consent, and the patient records/information were analyzed anonymously. The animal experimental procedures were carried out following the Guide for the Care and Use of Laboratory Animals of Xiangya Hospital, Central South University, with the approval of the Institutional Animal Ethics Committee.

### Statistical analysis

All experiments were independently repeated at least 3 times. Statistical analyses and charts were conducted by using IBM SPSS statistical software package 26 (IBM, NY, USA) and GraphPad Prism 8 (GraphPad, CA, USA). Student’s t test or Chi-square test were used to compare statistical difference between two groups. Kaplan-Meier survival analysis was used to compare NPC patient survival by the log rank test. Cox proportional hazards regression analyses were used to analyze the effect of clinical variables on patient survival. The Spearman rank correlation coefficient was used to determine the correlation between the two variables. All statistical tests were two-sided, and the *P* values <0.05 were considered to be statistically significant.

## Supplementary Material

Supplementary Figures

Supplementary Table 1
