# Supplementary material for: Flotillin-2 promotes cell proliferation via activating the c-Myc/BCAT1 axis by suppressing miR-33b-5p in nasopharyngeal carcinoma
Source: Aging (Albany NY). 2021 Mar 19;13(6):8078–94. doi: 10.18632/aging.202726 (PMC8034900; doi:10.18632/aging.202726)
Supplement: Supplementary Figures [file aging-13-202726-s001.pdf]

SUPPLEMENTARY FIGURES

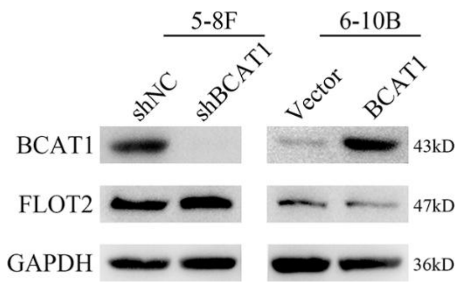

Supplementary Figure 1. FLOT2 is not regulated by BCAT1 in NPC. Western blot showing the level of c-Myc in 5-8F-shBCAT1, 6-10B-BCAT1 and control cells.

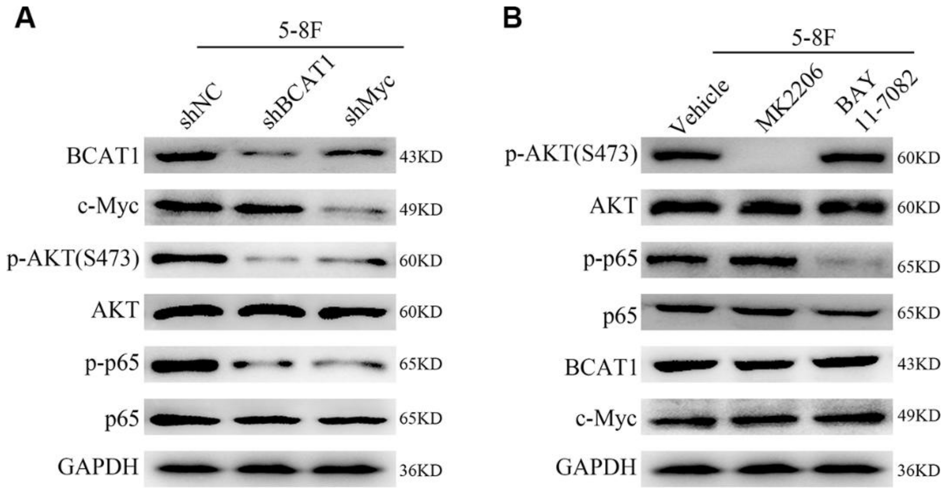

Supplementary Figure 2. c-Myc and BCAT1 knockdown inhibits activities AKT and NF-κB in NPC. (**A**) Western blot showing the level of BCAT1, c-Myc, p-AKT(S473), AKT, p-p65(S536) and p65 in 5-8F-shBCAT1, 5-8F-shcMyc and control cells. (**B**) Western blot showing the level of p-AKT(S473), AKT, p-p65(S536), p65, BCAT1, and c-Myc in 5-8F cells treated by MK2206(2.5μM) and BAY11-7082(5μM), respectively.

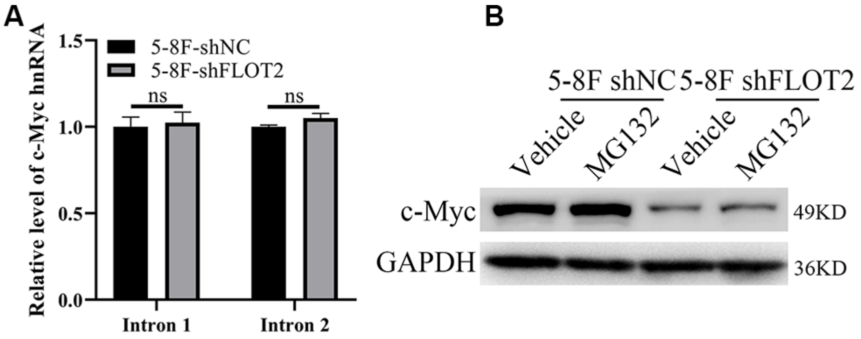

Supplementary Figure 3. FLOT2 does not regulate transcription and protein stability of c-Myc in NPC. (**A**) qPCR assay demonstrating the relative expression of c-Myc hnRNA in 5-8F-shFLOT2 and control cells using two pair of primers targeting intron-1 and intron-2, respectively. (**B**) Western blot showing the level of c-Myc in 5-8F-shFLOT21 and control cells treated by MG132 at 20mM. ns, no significant difference.
