# Supplementary material for: Flotillin-2 promotes cell proliferation via activating the c-Myc/BCAT1 axis by suppressing miR-33b-5p in nasopharyngeal carcinoma
Source: Aging (Albany NY). 2021 Mar 19;13(6):8078–94. doi: 10.18632/aging.202726 (PMC8034900; doi:10.18632/aging.202726)
Supplement: Supplementary Table 1 [file aging-13-202726-s002.pdf]

Supplementary Table 1. qPCR primers in this study.

Name	Primer sequence
miR-33b-5p	Cat.#, miRA1000051-1
5S	Cat.#, miRAN0001-1
BCAT1	F:5’-AGCCCTGCTCTTTGTACTCTT-3’
(NM_005504)	R:5’-CCAGGCTCTTACATACTTGGGA-3’
c-Myc	F: 5'-CCTACCCTCTCAACGACAGC-3
(NM_002467)	R: 5'-TTCCTCCTCAGAGTCGCTGC-3'
hnc-Myc Intron-1	F:5’- ATTTCTGACAGCCGGAGACG -3’
	R:5’- CCCAACACCACGTCCTAACA -3’
hnc-Myc Intron-2	F:5’- TGCTTGGGAATGTGCTTTGC -3’
	R:5’- AGCTGGGTTATGGCATGGAC -3’
GAPDH	F:5’- TGACTTCAACAGCGACACCCA -3’
(NM_002046)	R:5’- CACCCTGTTGCTGTAGCCAAA -3’
